# Direct structural evidence of Indian continental subduction beneath Myanmar

**DOI:** 10.1038/s41467-020-15746-3

**Published:** 2020-04-23

**Authors:** Tianyu Zheng, Yumei He, Lin Ding, Mingming Jiang, Yinshuang Ai, Chit Thet Mon, Guangbing Hou, Kyaing Sein, Myo Thant

**Affiliations:** 1 Key Laboratory of Earth and Planetary Physics, Institute of Geology and Geophysics, Chinese Academy of Sciences, Beijing, 100029 China; 2 CAS Center for Excellence in Tibetan Plateau Earth Sciences, Chinese Academy of Sciences, Beijing, 100101 China; 3 Key Laboratory of Continental Collision and Plateau Uplift, Institute of Tibetan Plateau Research, Chinese Academy of Sciences, Beijing, 100101 China; 4 Department of Geology, Dagon University, Yangon, Myanmar; 5 Myanmar Geosciences Society, Yangon, Myanmar; 6 Department of Geology, University of Yangon, Yangon, Myanmar; 7 Myanmar Earthquake Committee, Hlaing Universitiesʼ Campus, Hlaing Township, Yangon, 11052 Myanmar

**Keywords:** Geophysics, Seismology, Tectonics

## Abstract

Indian continental subduction can explain Cenozoic crustal deformation, magmatic activity and uplift of the Tibetan Plateau following the India-Asia collision. In the western Himalayan syntaxis and central Himalaya, subduction or underthrusting of the Indian Plate beneath the Eurasian Plate is well known from seismological studies. However, because information on the deep structure of the eastern Himalayan syntaxis is lacking, the nature of the Indian subduction slab beneath Myanmar and the related tectonic regime remain unclear. Here, we use receiver function common conversion point imaging from a densely spaced seismic array to detect direct structural evidence of present-day Indian continental subduction beneath Asia. The entire subducting Indian crust has an average crustal thickness of ~30 km, dips at an angle of ~19°, and extends to a depth of 100 km under central Myanmar. These results reveal a unique continental subduction regime as a result of Indian-Eurasian continental collision and lateral extrusion.

## Abstract (web summary)

Indian continental subduction can explain crustal deformation, magmatic activity and uplift of the Tibetan Plateau following collision, however, the nature of the Indian subducting slab beneath Myanmar and the related tectonic regime remain unclear. Here, the authors present direct structural evidence of present-day Indian continental subduction beneath Asia.

## Introduction

**Fig. 1:**
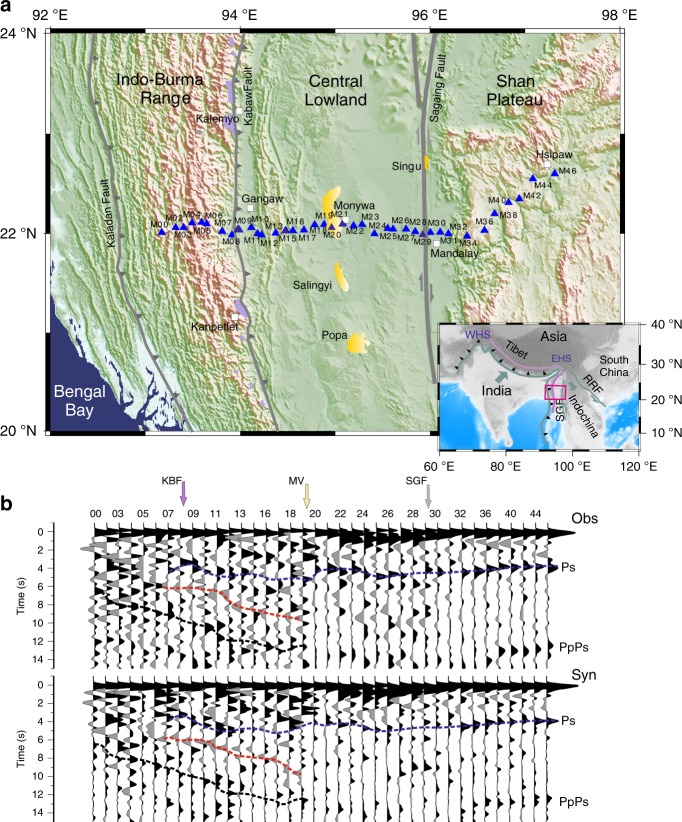
Topographic map of the study region showing simplified tectonic divisions, distribution of seismic stations, and cross-sections of P receiver functions. **a** Map of the convergent system of the Indian and Asian plates in the Myanmar region. Each seismic station used in this study is shown with a blue triangle. The yellow areas mark the outcrops of Late Cretaceous to Miocene volcanic rocks, and the purple areas mark ophiolites in the IBR. The topographic data are derived from the ETOPO1 database^40^. The inset shows the location of the study region in the India–Asia collision system. **b** Cross-sections (from west to east) of stacked P receiver functions from data (Obs) and synthetic (Syn) images. Traces are plotted in a time window between −1 and 15 s with time-zero aligning with the onset of the P-wave. The amplitudes of the traces are normalized by the maximum amplitude of all traces. Station numbers are labeled at the top of the plots for ease of reference. Dashed lines indicate the converted phases from the Moho (blue), the top interface (red), and the bottom interface (black) of a dipping structure recognized by waveform inversion. *EHS* eastern Himalayan syntaxis, *WHS* western Himalayan syntaxis, *KBF* Kabaw fault, *MV* Monywa volcanoes, *SGF* Sagaing fault, *RRF* Red River fault.

The fate of the Indian Plate during continental collision with Asian terranes is one of the core issues for understanding continental subduction following continent–continent collision and resultant effects. Diverse and complex tectonic activity has taken place along the India–Asia collision zone since the Eocene. In the central Himalaya, the underthrusting of the Indian Plate beneath the Tibetan Plateau has been well revealed by seismic imaging^1,2^. Through tectonic reconstructions, the high-velocity anomalies in the upper mantle identified by seismic tomography have been interpreted as evidence of Indian continental lithosphere subduction^3,4^. In the western Himalayan syntaxis (WHS), two converging continental subduction zones have been reported to extend to a depth of ~400 km, with a south-dipping 10–15 km-thick low-velocity zone proposed as the subducting Eurasian continental lower crust beneath the Pamir and a north-dipping Indian slab beneath the Hindu Kush^5–8^. Myanmar, situated along the obliquely colliding eastern margin of the Indian Plate (Fig. 1A), connects the ongoing India–Asia collision along the Himalayas in the north to the newly formed oceanic crust in the Andaman Sea in the south^9^. The seismicity outlines a Wadati-Benioff zone beneath Myanmar^10–12^ that approximately coincides with a broad high-velocity structure revealed by regional and global tomographic studies, hinting at ongoing subduction^4,13,14^. However, given the lack of detailed geometry and structures, the nature of the ongoing subduction and the related tectonic regime remain unclear.

Following the events in the Late Cretaceous-Paleogene, the Myanmar region can be divided into three physiographic units (Fig. 1A). In the east, the Shan Plateau comprises Cambrian-Jurassic sequences, a slate belt, and the Mogok metamorphic belt intruded by Late Cretaceous-Miocene granites, which appear to correlate with the rifting of the Sibumasu terrane from Gondwana and subsequent docking with the Indochina terrane during the Mesozoic^15^.

In the middle, the Central Lowland comprises Mesozoic and Cenozoic volcanic arcs and related forearc and back-arc basin deposits. Mitchell^16^ infers a latest Early Cretaceous age for the initiation of Burma volcanic arc magmatism related to Tethyan ocean subduction. Geochemical data indicate that late Cenozoic volcanic rocks from the Monywa and Popa areas erupted in two distinct stages, i.e., mid-Miocene and Quaternary^17^. Most mid-Miocene volcanic rocks have intermediate compositions with high-K calc-alkaline affinity, whereas the Quaternary volcanic rocks exhibit mafic compositions with high-K calc-alkaline and shoshonitic affinities^17^, implying a subduction setting.

In the west, the Indo-Burma Range (IBR) is a fold-and-thrust belt composed of ultramafic rocks, schists, pillow lavas, and Triassic-Eocene sedimentary rocks^18^. The Western Ophiolitic Belt in Myanmar follows the eastern margin of the IBR and the outer Andaman island arc. The ophiolites of Middle Jurassic-Early Cretaceous age appear to be related to the suture zone created by the closure of the Neo-Tethys Ocean^18,19^. During the Neogene, the IBR is considered to have formed as an accretionary wedge linked to the eastward subduction of the continental crust beneath the Bengal Basin^18^. The joint study of multichannel seismic reflection, gravity, magnetic, and bathymetric data in the Bay of Bengal south of 18°N implies that the oceanic crust of the Indian Plate is steeply subducting beneath the Burma microplate^20^. The shear wave velocity structure beneath the Bay of Bengal estimated from the surface wave data indicate that the crustal thickness increases from south to north and that the crustal velocity decreases from a higher oceanic crust-like value at the southern end to a lower continental crust-like value at the northern end^21^.

To gain insights into the previously unknown deep processes of subduction evolution beneath Myanmar and their relations to the India–Tibet subduction system, the detailed geometry and the nature of the subducting plate constrained by structural imaging are crucial. We implemented the China–Myanmar Geophysical Survey in the Myanmar Orogen (CMGSMO) project to deploy the first portable seismic array in north-central Myanmar in cooperation with the Myanmar Geosciences Society (MGS). Here, we present a receiver function common conversion point (CCP) image^22^ beneath the main-line profile of the CMGSMO crossing Myanmar in the E–W direction. The revealed continental subduction structures have an average crustal thickness of ~30 km, dip at an angle of ~19°, and extend to a depth of 100 km under central Myanmar, largely different from those in the WHS. Incorporating our results with the sideways extrusion tectonics of the Asian lithosphere, we propose a scenario of a geodynamic regime in a continental subduction system following continental collision with lateral extrusion.

## Results

### Seismic data and section features of receiver functions

Seismic data obtained from a dense seismic array (the Central Myanmar Seismic Profile, CMSP) comprising 38 temporary stations were used to image the structures of the crust and upper mantle in Myanmar. From June 2016 to January 2018, the CMSP was temporarily deployed over a distance of ~450 km in an E–W direction along latitude ~22° N with station spacing of 10–20 km across the IBR (stations M00-M08), the Central Lowland (stations M09–M29), the Sagaing fault, and the Shan Plateau (stations M30–M46) (Fig. 1A). We employ receiver function imaging to infer the velocity structure beneath the CMSP. The records of teleseismic events with magnitudes larger than 5.8 and epicentral distances in the range of 30°–90° are converted to P receiver functions. The back azimuths of events range over the whole space, with most falling within the range of 30°–140° (Supplementary Figs. 1 and 2). The piercing points for P-to-S converted phases at 100 km depth are distributed north and south of the CMSP >50 km apart. The back-azimuth distribution on the northern side is significantly better than that on the southern side. For the western section of the CMSP (stations M00–M19), receiver functions coming from the north (in the back-azimuth ranges of 0°–92° and 270°–360°) are used to determine the structure with depths of 0–120 km by CCP imaging. The data in the back-azimuth range of 0°–92° are stacked and used in waveform inversion. For the eastern section (stations M20–M46), the imaging object is normal crust with a thickness <40 km, and the receiver functions of full back azimuths are used to image the crustal structure (Supplementary Table 1). The stacked receiver functions at each station are ranked along the imaged profile from west to east (Fig. 1B). The first-order feature of the receiver function cross-sections is the distinct difference in waveforms between the western and eastern parts. The Ps phases converted from the Moho and the following PpPs phases emerge in the eastern part. In the western part, in addition to the Ps phases from the Moho, we find two eastward-dipping running phases with a negative phase above and a positive phase below emerging at ~5–13 s, corresponding to depths of ~40–100 km.

### Common conversion point stacking and structural imaging

**Fig. 2:**
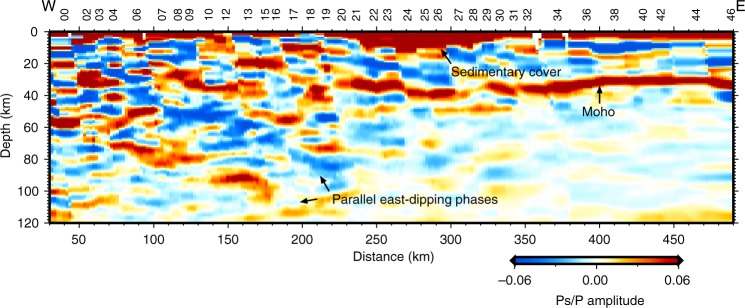
Initial common conversion point (CCP) image along the Central Myanmar Seismic Profile. The CCP image is generated using a prior one-dimensional model as the migration model. Red colors denote positive receiver function amplitudes as indicated by the color bar, and blue colors denote negative amplitudes.

Taking advantage of the energy concentration from the P-S converted phases, the migration and stack imaging of receiver functions in the depth domain is a robust method to directly identify velocity discontinuities in the crust and upper mantle. We apply the CCP stacking of radial receiver functions to investigate the structure of the crust and upper mantle. First, we obtain the CCP image using a prior model as a migration model. The prior model is a modified preliminary reference Earth model (PREM), in which the crustal part is replaced by an average one-dimensional model for Yunnan (eastern adjacent region). From the initial CCP image along the CMSP (Fig. 2), we can roughly identify the dominant sedimentary cover beneath stations M21–M30 and the Moho interface in the depth range of 30–40 km. Notable features are two parallel east-dipping phases (negative phase above and positive phase below) appearing in the upper mantle beneath stations M00–M19.

However, the multiple PpPs and PsPs+PpSs waves generated by shallow structures seriously interfere with identifying the real velocity discontinuities. Determining the properties of these interfaces, in addition to the Moho interface, is difficult. Here, we apply a synthetic test of CCP imaging to distinguish reliable intracrustal and upper mantle interfaces. Based on the velocity models resulting from stepwise waveform inversion from shallow to deep, we generate a set of synthetic CCP images from synthetic receiver functions and compare them with the observed CCP image from the observed receive functions.

**Fig. 3:**
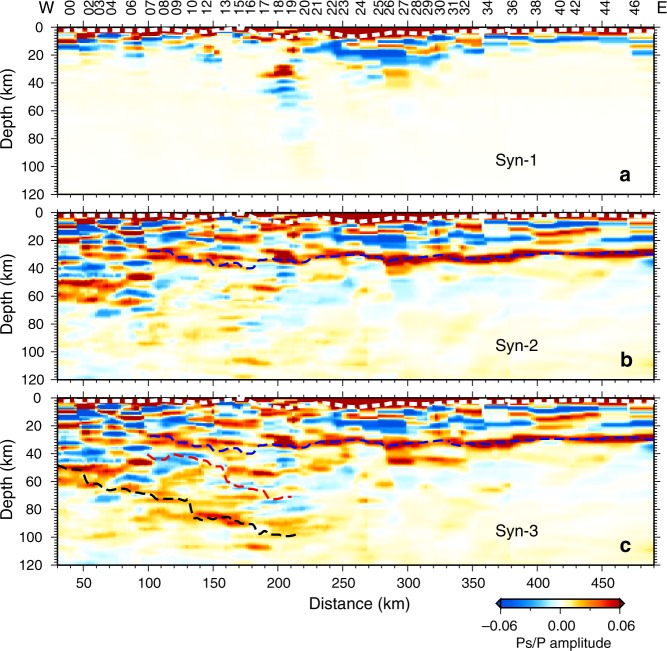
Synthetic test of the common conversion point (CCP) imaging. **a** Syn-1 is calculated based on the model with a homogeneous medium underlying the sedimentary cover resulting from the waveform inversion. **b** Syn-2 is calculated based on the model with a homogeneous mantle medium underlying the crustal models above ~35 km resulting from the waveform inversion. **c** Syn-3 is the synthetic CCP image calculated based on the model with a dipping structure in the upper mantle. To show the structural features of the imaged crust and mantle wedge more clearly, a lighter color bar is used in the unstudied part of the mantle. Dashed lines in the CCP image mark the locations of velocity discontinuities, including the basement (white), Moho (blue), top interface (red) and bottom interface (black) of the dipping structure from the observed CCP image (Fig. 4A).

**Fig. 4:**
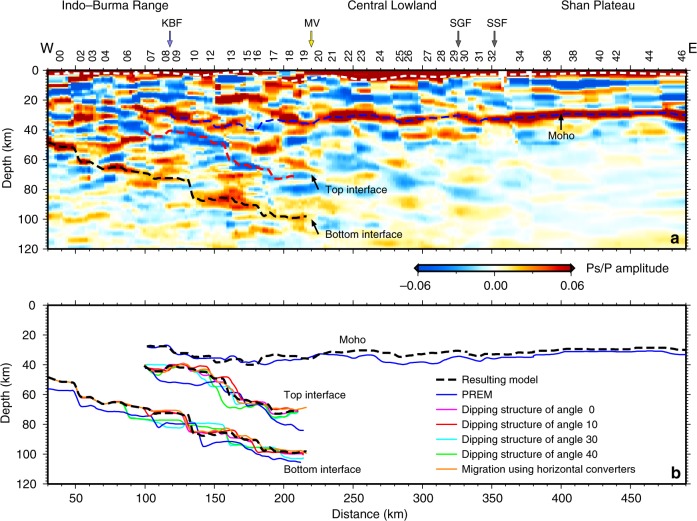
Resulting common conversion point (CCP) image and reliability analysis. **a** Resulting CCP image along the seismic profile CMSP. Dashed lines in the CCP image mark the locations of velocity discontinuities, including the basement (white), Moho (blue), top interface (red), and bottom interface (black) of the gently dipping structure. KBF: Kabaw fault; MV: Monywa volcanoes; SGF: Sagaing fault; SSF: Shan Scarp fault. **b** Comparison of the interface depths identified from CCP images with different migration models. Various colors mark the interface of the Moho and the top and bottom interfaces of the gently dipping structure obtained from different models. Dashed lines mark the interfaces from the resulting CCP imaging.

We first estimate the velocity structure of the sedimentary cover by waveform inversion with constraints on the thickness of sedimentary strata revealed by the observed CCP profile. We then calculate synthetic CCP image Syn-1 (Fig. 3A) based on the synthetic receiver functions generated by the velocity models of a PREM underlying the sedimentary cover models. Large-amplitude positive and negative signals from the surface to a depth of >15 km are observed (Fig. 3A), even though the sedimentary cover is <7.5 km thick (indicated by the white dashed line in Fig. 3). The signals below the sedimentary cover in Fig. 3A, which are generated by multiples, clearly cannot be identified as velocity discontinuities. At the same time, the signals in the upper part of the crust that appear in the observed CCP image (Fig. 4A) but not in the Syn-1 image should be considered intracrustal interfaces.

The strong positive phase at a depth of ~30 km in the observed CCP image is the converted phase from the Moho. To distinguish the multiples from intracrustal interfaces and real interfaces in the upper mantle, we iteratively run the above process. The Syn-2 image (Fig. 3B) is calculated based on the synthetic receiver functions generated by the velocity models of a homogeneous mantle medium underlying the crustal models resulting from the waveform inversion. As shown in Fig. 3B, despite the well-constructed crustal structure, no dipping structure with two parallel phases (negative phase above and positive phase below) appears in the upper mantle beneath stations M00–M19, which is different from the observed CCP image. This result indicates that the two parallel phases represent velocity discontinuities in the upper mantle, rather than multiples generated by intracrustal interfaces. Syn-3 (Fig. 3C) is the synthetic CCP image obtained from the model with a dipping structure in the upper mantle.

After the synthetic test of CCP imaging, the resulting best-fit velocity models derived from waveform inversion of receiver functions at each station (Supplementary Fig. 3) are merged as a quasi-two-dimensional migration model to replace the prior model in the CCP imaging. As the cross-sections of the receiver functions (Fig. 1B) hint at a dipping structure, a dipping converter at the dipping layered structures^7,23^ is also utilized in the migration. Finally, we obtain the refined CCP image along the CMSP (Fig. 4A). Three strong running phases, including a positive phase at a depth of ~30–35 km and two eastward-dipping phases (a negative phase above and a positive phase below) in the depth range of ~40–100 km in the western part are identified in the CCP image and are real velocity discontinuities, not the signals of multiple waves from shallow structures.

The crustal image revealed by CCP stacking (Fig. 4A) effectively divides the crust into three parts with different features roughly corresponding to the surface tectonic units. The Shan Plateau in the eastern part of the section (stations M30–M46) features a relatively gentle Moho topography at a depth of ~30 km and then deepens westward to 34–36 km. The Moho is uplifted ~2 km and is weakened beneath the Sagaing fault (stations 28–29), indicating that this fault may extend through the crust. Beneath the Central Lowland (stations M09–M29), the Moho topography is rugged, and the crust–mantle transition is sharp in the center and diffuse on both sides. Under the IBR, a strong positive velocity discontinuity appears at a depth range of 50 km (beneath station M00) −70 km (beneath station M06). This discontinuity may indicate the Moho interface of the subducting Indian Plate underlying a 20–30 km-thick accretionary wedge.

A prominent eastward-dipping structure is imaged beneath the western CMSP. Two parallel interfaces tilt down 19° with a thickness of 23–39 km (average thickness of 30 km). The top interface of the dipping structure is identified by the negative running phase in the CCP image, and the bottom interface is identified by the positive running phase between depths of ~70 and 100 km.

### Reliability analysis of CCP imaging of the gently dipping structure

In our study, a gently dipping structure (~19°) is observed in the CCP image. Previous research has indicated that CCP stacking with a flat-layer migration model would be valid if the object to be reconstructed is horizontal or slightly inclined (<30°)^7^. Here, a dipping converter with an inclined structure^7^ is also utilized in migration for more accurate recovery of interfaces than that obtained by using a horizontal converter. We estimate the reliability of the resulting CCP imaging by synthetic tests. We compare the Moho depths of the overlying plate and the top and bottom interface depths, thicknesses, and dip angles of the gently dipping structure obtained from the resulting CCP images with those from the CCP images based on different migration models (Fig. 4B, Supplementary Table 2). The geometry of the interface is defined by the depth of the local maximum amplitude in the CCP images. The dip angle of the dipping structure is measured based on the linear regression line of the top interface. The results from the resulting CCP imaging are also shown in Fig. 4B. The test models include the migration models with different dipping layered structures, a modified PREM without a dipping structure in the upper mantle, and the best-fit velocity model as the migration model but a horizontal converter applied in the migration. The comparison of the interface depths and thicknesses between the tested and the resulting CCP images is used to estimate the reliability of the seismic imaging. The standard deviations of interface depths from models with dip angles <20° are <2 km at the top interface of the dipping structure and <1 km at the bottom interface of the dipping structure. Imaging based on migration using horizontal converters produces standard deviations similar to those of the models with low dip angles. The imaged dip angle from migration models with dip angles larger than 30° is 21°–22° and that from other migration models is 19°–20°. This analysis indicates that the dipping structure with a dip angle of ~19°–20° is a reliable result. The standard deviation of the Moho depth from PREM is 2.6 km, and the standard deviation of the interfaces of the dipping structure is 5.9 km. The standard deviations of the top interface with dip angles larger than 30° are ~3 km, and those of the bottom interface are 3–5 km. These results indicate the impact of setting a proper migration model in CCP imaging. Despite the large standard deviations obtained from PREM and the models with dip angles larger than 30°, prominent eastward-dipping structures are imaged in all of the tests. The reliability test of CCP imaging demonstrates that the gently dipping structure beneath the CMSP is not an artifact.

## Discussion

**Fig. 5:**
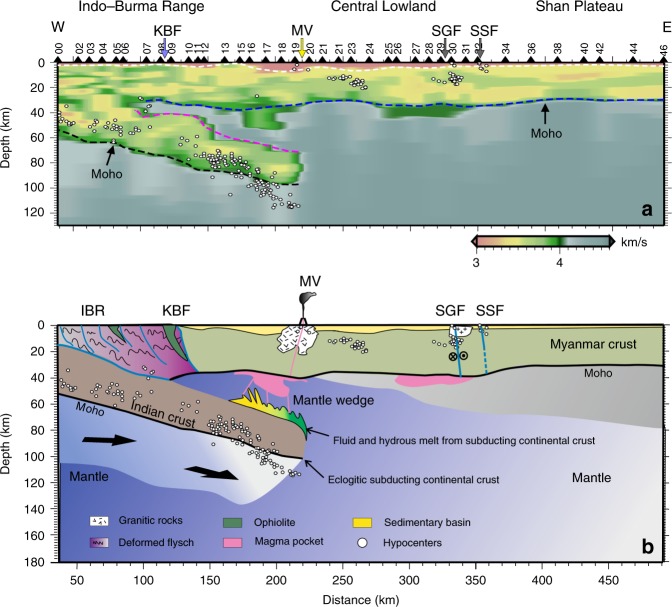
Shear wave velocity structure and related schematic model. **a** Shear wave velocity structures of the crust and uppermost mantle compiled from the velocity models resulting from waveform inversion beneath each station along the CMSP. The values of shear velocity are indicated by the color bar at the bottom of the map. The white dashed line represents the basement interface, and the blue dashed line indicates the depth of the Moho along the profile. The dipping crustal segment in the mantle is bounded by the magenta and black dashed lines. White dots denote the newly located shallow and intermediate-depth earthquakes, and only the earthquakes located within 0.25° of the profile are shown here. **b** Schematic model of Indian continental subduction tectonics beneath Myanmar. White dots denote the newly located shallow and intermediate-depth earthquakes, which are the same as those in **a**. KBF: Kabaw fault; MV: Monywa volcanoes; SGF: Sagaing fault; SSF: Shan Scarp fault.

We show the shear wave velocity structure of the CMSP (Fig. 5A), which is compiled from the velocity models resulting from the waveform inversion beneath each station (Supplementary Fig. 3), to further understand the structural characteristics related to subduction (Fig. 5B). In the IBR, the accretionary wedge is 20–30 km thick overlying the subducting Indian Plate and is characterized by high- and low-velocity interlayers. In the Central Lowland, a continental crustal structure consisting of the upper crust and the lower crust has shear wave velocities (Vs) of 3.5–3.6 km/s and 3.7–3.9 km/s, respectively, and is covered by a sedimentary sequence with a maximum thickness of 7 km. Beneath the Shan Plateau, the intracrustal interfaces are gently elongated, and the low-velocity zones are located near the crust–mantle boundary.

In particular, our imaging results also show that a low-velocity structure with a dip angle of ~19° extends to a depth of 100 km beneath the IBR and the western Central Lowland. The large thickness of the eastward-dipping low-velocity structure located beneath the Myanmar crust, which is >30 km thick, implies that this layer is not oceanic crust. The upper part of the dipping layer has shear wave velocities of 3.5–3.8 km/s, and the lower part has shear wave velocities of 3.7–4.0 km/s, indicating an intermediate mineral composition. We thus identify the dipping layer as the Indian continental crust.

Shallow and intermediate-depth earthquakes occurring during June 2016–January 2018 in Myanmar between latitudes 21.75° and 22.25° N are also shown in Fig. 5. We pick these local earthquakes from seismic data for the entire CMGSMO network (Supplementary Fig. 1) and relocate the epicenters by the double-difference method^24^. The location errors in longitude, latitude and depth are 2.9, 3.3, and 5.4 km, respectively. We find that shallow earthquakes are located beneath the Central Lowland and the Sagaing fault. The intermediate-depth earthquakes are mainly concentrated along the lower part of the subducting crust at depths of 70–120 km. The deep front end of the intermediate-depth earthquake cluster extends slightly beyond the lower boundary of the imaged subducting crust. Eclogitization of a metastable subducting Indian continental lower crust in the presence of water may dominate the occurrence and distribution of intermediate-depth earthquakes shallower than 100 km^25^. The deep earthquakes beyond the subducting crust may hint at other embrittlement mechanisms in the lithospheric mantle under ductile conditions. We suspect that the shear instability induced by breakoff of the subducted slab and thermal runaway might be a likely mechanism^26,27^, analogous to those in the Hindu Kush^8,28^. Further geological and geophysical investigations on the eclogitization of the subducting Indian continental crust are necessary in the future.

Along the CMSP, for the first time, the detailed velocity structures of subducting continental crust and overlying mantle wedge are imaged. Informed by petrological research in Dabie^29^, we interpret the low shear wave velocity zones in a depth range of 40–60 km in the mantle wedge (Fig. 5A, beneath stations M13–M19) as ponding mantle magmas (Fig. 5B). Studies of oceanic plate subduction indicate that arc magmas are formed by the partial melting of the mantle wedge, which is metasomatized by fluids or melts released from the underlying subducted oceanic slab. A similar process for continental subduction was suggested by a detailed petrological study of Maowu garnet orthopyroxenites^29^ in Dabie, a continental subduction-collision zone. Crustal rocks were subducted into the deeper part of the cold mantle wedge zone in a depth range of ~70–150 km, dehydration reactions occurred, and a large amount of fluid was released from the lower margin of the cold mantle wedge. The released fluid might also have migrated into the overlying, hotter part of the mantle wedge in a depth range of ~40–100 km, resulting in a high degree of partial melting and the formation of arc magmas. The ponding mantle magmas beneath the CMSP may have played a role in the generation of late Cenozoic igneous activity.

The present geometry of the Indochina block has been suggested to be a consequence of collision-driven extrusion^10,30,31^. Based on global tomographic images, Replumaz et al.^4^ interpreted the high-speed anomaly to a depth of 400 km beneath Myanmar as a slab of Indian lithosphere pushed away from the frontal collision by the extrusion of Indochina. A well-defined continental slab revealed by our new seismic imaging indicates ongoing subduction of Indian continental crust beneath Myanmar to a depth of ~100 km. We infer that the slab is part of the Indian continent. This part of the Indian continent was most likely located initially on the northeast margin of India and is presently subducting under Myanmar following the clockwise rotation of Indochina around the eastern Himalayan syntaxis (EHS).

The differences in the WHS and EHS subduction structures provide us with important information to understand continental subduction after continental collision. At the WHS, seismological studies of the Hindu Kush-Pamir orogenic system provide a structural image of continental collision linked to two opposing intracontinental subduction zones: northward subduction of Indian lithosphere beneath the Hindu Kush and southward subduction of Asian lithosphere beneath the Pamir^6–8^. The subduction zones in the WHS and EHS are dramatically different in terms of slab steepness and subduction depth. The subducting Indian continental crust underneath the Hindu Kush reaches ~180 km with dip angles of ~65–80°^8,28^, and the subducting Eurasian continental crust beneath the Pamir extends to >150 km with dip angles increasing to subvertical^6,7^. Both are steeper and deeper than the subducting slab underneath Myanmar (~19° and ~100 km). Another striking difference between the slab structures is lower crust subduction beneath the Pamir, mid-to-lower crust subduction beneath the Hindu Kush in the WHS^7,28^ and entire crust subduction beneath Myanmar in the EHS. The subducting crustal thickness of 23–39 km observed beneath Myanmar indicates that upper crustal material has not been mechanically decoupled from the lower crust during continental subduction. In the regime of continental collision, geodynamic modeling^32^ has shown that the subduction of the entire crust is possible only if the upper crust and lower crust are fully coupled.

We propose that the discrepancies in continental subduction between the WHS and EHS can be primarily attributed to the sideways extrusion of Asian lithosphere. The asymmetry of collisional deformation suggests that the continental lithosphere in western Eurasia offers more resistance to lateral motions than the subduction zone along the Pacific and Indonesian margins and Asian lithospheric materials are extruded along the eastern margin of the Indian Plate^30,31^. Accordingly, asymmetric continental subduction tectonics in the India–Asia collision zone is expected. The subduction occurring in a compressional regime in the WHS is represented by two converging subduction zones with the lower and possibly middle crust subducted^7,28^. The continental collision is weakened by shearing-derived extrusion in the EHS, which decreases the resistance to subduction and leads to subduction of the entire continental crust. The difference in the depths of continental subduction beneath the WHS and EHS may be partly caused by the difference in integrity of the subducting continental crust^33^.

In this study, the subducting continental crust is explicitly explored by seismic observation and imaging. We delineate a continental subduction regime resulting from continental collision with lateral extrusion. The continental collision is weakened by the lateral extrusion of the Tibetan lithosphere, and the decreased resistance to subduction enables subduction of the entire continental crust. This tectonic pattern defines a unique geodynamic regime of a subduction system following continental collision with lateral extrusion. As a result, the primary characteristics of crust–mantle interactions and the formation of arc magmatism in continental subduction zones have been preserved. Our study of continental subduction beneath Myanmar is based on evidence from crustal and upper mantle structures, and exploration for petrologic and geochemical evidence of continental subduction is necessary in the future.

## Methods

### Receiver function imaging

We apply the CCP stacking of receiver functions to investigate the velocity structure of the crust and upper mantle. Some efforts have been made to improve the capability of structural imaging^34,35^. The velocity models derived from waveform inversion of receiver functions at each station are used as the migration model in the CCP imaging. A dipping converter with an inclined layered structure is utilized in migration. The synthetic modeling of CCP images is used to determine the structural constituents of the crust and upper mantle and to distinguish the real interfaces from multiple phases.

Receiver function imaging was carried out in five steps. First, we produced the observed CCP images using a regional average velocity model obtained from a previous study as the migration model. Second, we inferred the 1-D velocity models via waveform inversion of the stacked receiver functions for each station using constraints on the interface depths from the observed CCP image (Supplementary Fig. 3). Third, we calculated the synthetic CCP images (Syn, for example, in Fig. 3). The synthetic receiver functions were computed using the same ray paths as the observations and the 1-D velocity models resulting from the waveform inversion. Fourth, by comparing the observed and synthetic CCP images (Figs. 3 and 4A), we distinguished real discontinuities from multiple phases, systematically adjusted the inversion spaces of the velocity models at each station, and then executed steps 2, 3, and 4 repeatedly. The velocity discontinuities identified via the previous synthetic CCP modeling were used to constrain the subsequent waveform inversion. We also produced an improved observed CCP image with the resulting velocity models as migration models. Fifth, the final confirmations of the velocity structures were obtained based on an optimal match between the synthetic and observed CCP images. Dual constraints on the CCP image and seismic waveform significantly enhanced the reliability of the receiver function imaging.

### Three principal technique components

Receiver function offsetting and stacking in the depth domain, synthetic receiver function computation, and waveform inversion are three principal technique components. Receiver functions were constructed using a time-domain maximum entropy deconvolution method^36^. The data were band-pass filtered with corner frequencies of 0.05 Hz and 4 Hz. Waveforms were windowed from 20 s before to 100 s after the onset of the P-wave. A Gaussian parameter of 5.0 and a water level of 0.0001 were adopted in the deconvolution. The data selection process yielded >100 receiver functions from most stations (Supplementary Table 1). The CCP stacking method^22^ was used to calculate the depth domain stacking image. A dipping converter with dipping layered structure^23^ was also utilized in migration, in which the ray paths were computed to satisfy Snell’s law at a converter with a defined inclination angle. Synthetic receiver functions were calculated by the reflection matrix method following Kennett (1983)^37^.

The inversion was performed using an adapted hybrid global inversion method^38,39^ based on structural constraints from the CCP images to construct the velocity model. In our receiver function inversion, the best fit between the synthetic receiver function and the observed one is searched by minimizing the objective function, which measures the degree of coincidence of both waveforms and amplitudes between observations and synthetics. Supplementary Fig. 3 shows the waveform comparison between the stacked receiver functions from the data and the synthetic receiver functions and the best-fit shear wave velocity model at each station. The waveforms of the synthetic receiver functions fit closely with the observed waveforms with respect to both arrival times and relative amplitudes beneath most of the stations. Major structural characteristics can be coherently detected.

### Reliability analysis

We measured the differences of interface depth *Y*_*j*_*(x*_*i*_*)−Y*_*0*_*(x*_*i*_*)* between the CCP images of different test models (*Y*_*j*_) and the resulting CCP image (*Y*_*0*_) for each pixel along the *x* axis, where *Y* represents the value of Moho depth, top interface depth, bottom interface depth, and thickness of the dipping structure along the section, *x* is the surface location of the section with a sampling interval of 1 km. Here *j* = 0 for the best-fit model, and *j* = 1, 2, 3, 4, 5, 6 for test models. The standard deviation and maximum deviation within 90% confidence intervals are listed in Supplementary Table 2. The estimation of deviations reflects the depth resolution of velocity interfaces.

## Supplementary information

Supplementary Information

## Data Availability

Seismic data from the CMGSMO project are managed and deposited in the Seismic Array Laboratory, IGGCAS (10.12129/IGGSL.Data.Observation, http://www.seislab.cn). The seismic receiver functions for this study can be downloaded via ftp://159.226.119.161/data/CMGSMO-1/RF_mainprofile.

## Code availability

The CCP code is available at http://www.eas.slu.edu/People/LZhu/home.html. The hypoDD code used to relocate earthquakes is available at https://www.ldeo.columbia.edu/~felixw/hypoDD.html. The GMT code used to create the figures is available at http://www.soest.hawaii.edu/gmt. The other codes mentioned in this paper are available to interested researchers upon request.
