# Supplementary material for: Direct structural evidence of Indian continental subduction beneath Myanmar
Source: Nat Commun. 2020 Apr 23;11:1944. doi: 10.1038/s41467-020-15746-3 (PMC7181713; doi:10.1038/s41467-020-15746-3)
Supplement: Supplementary file 1 — Supplementary Information [file 41467_2020_15746_MOESM1_ESM.pdf]

Direct structural evidence of Indian continental subduction beneath Myanmar

Tianyu Zheng[1], Yumei He*[1,2], Lin Ding[2,3], Mingming Jiang[1,2], Yinshuang Ai[1,2], Chit Thet Mon[1,4], Guangbing Hou[1], Kyaing Sein[5], Myo Thant[6]

[1]Key Laboratory of Earth and Planetary Physics, Institute of Geology and Geophysics, Chinese Academy of Sciences, Beijing, 100029, China

[2]CAS Center for Excellence in Tibetan Plateau Earth Sciences, Chinese Academy of Sciences, Beijing 100101, China.

[3]Key Laboratory of Continental Collision and Plateau Uplift, Institute of Tibetan Plateau

Research, Chinese Academy of Sciences, Beijing 100101, China

[4]Department of Geology, Dagon University, Myanmar

[5]Myanmar Geosciences Society, Myanmar

[6]Department of Geology, University of Yangon, Myanmar

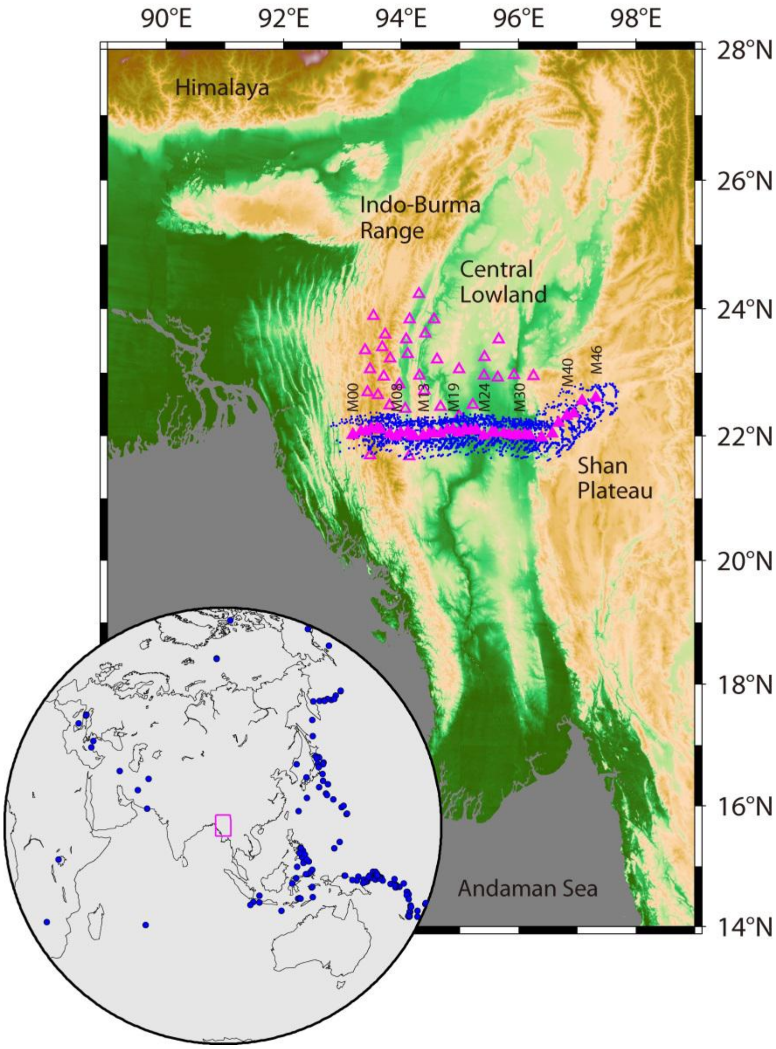

Supplementary Figure 1 Topographic map of Myanmar and adjacent areas and the distribution of seismic stations

Magenta bold triangles represent the seismic stations used in this study for receiver function imaging. Blue dots mark the piercing points at a depth of 100 km for P-to-S converted phases. The magenta open triangles mark the locations of 32 off-line stations, which were also used in earthquake location. The topographic data are derived from the ETOPO1 database[1]. The inset shows the distribution of teleseismic events at epicentral distances between 30° and 90° with body wave magnitudes of ≥5.8.

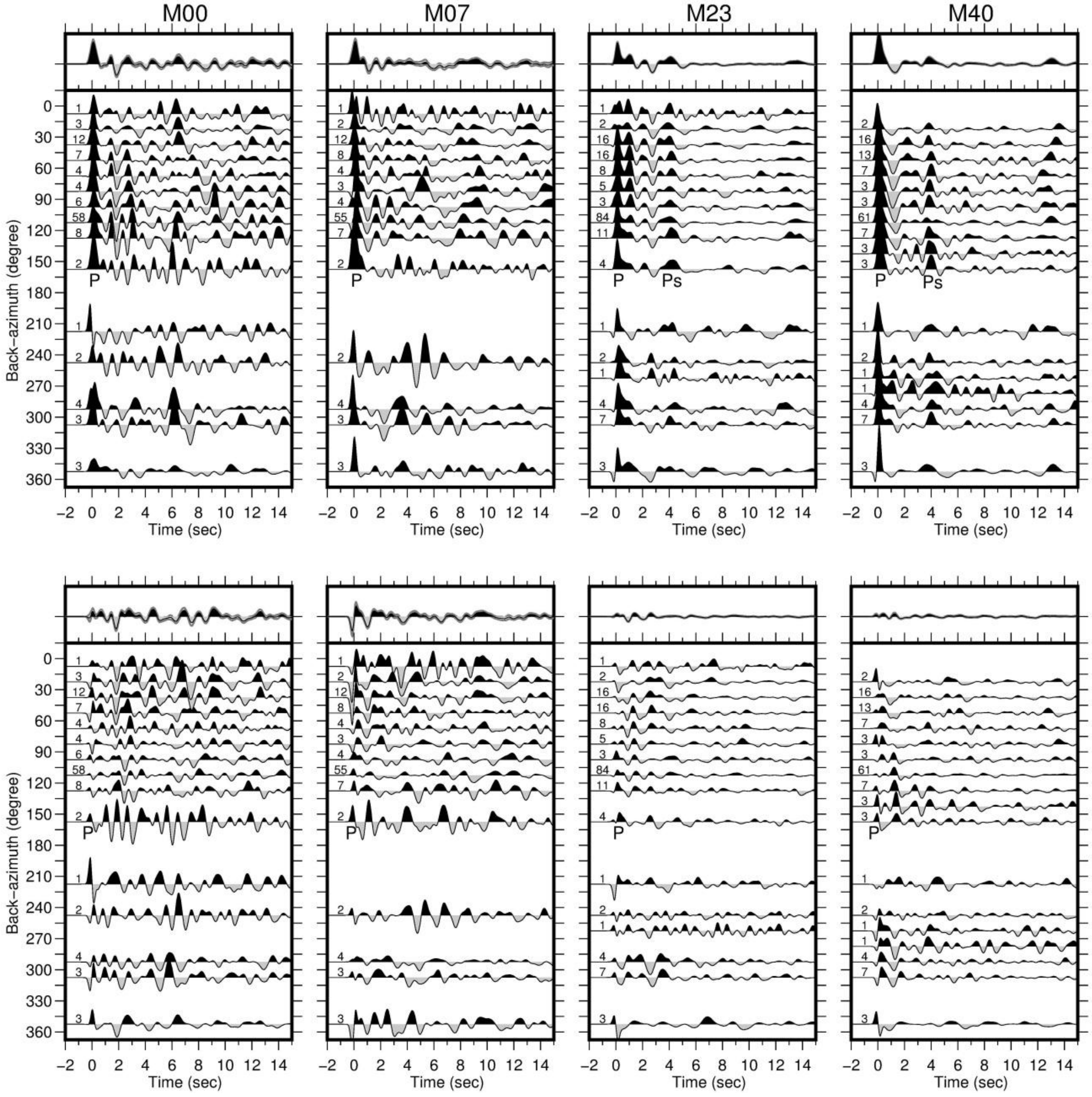

Supplementary Figure 2 Radial (top) and Tangential (bottom) receiver functions stacked at back-azimuth intervals of 15° at stations M00, M07, M23 and M40

The number at the left indicates the number of stacked traces in each bin. On the top, stacked traces (solid lines) are shown. At stations M00 and M07, only receiver functions in the range of northeast back-azimuth (0° - 92°) are summed. All receiver functions are summed at stations M23 and M40. 95% confidence levels (gray lines) estimated by the bootstrapping method are also shown here. The amplitudes of the traces are normalized in each panel. The clear variations in the radial waveforms with azimuth are observed from the receiver functions at stations M00 and M07. We can also observe strong energy on the transverse component at stations M00 and M07, where the dipping structure exists at depth, and less energy at stations M23 and M40, where the

structure is more 1-D.

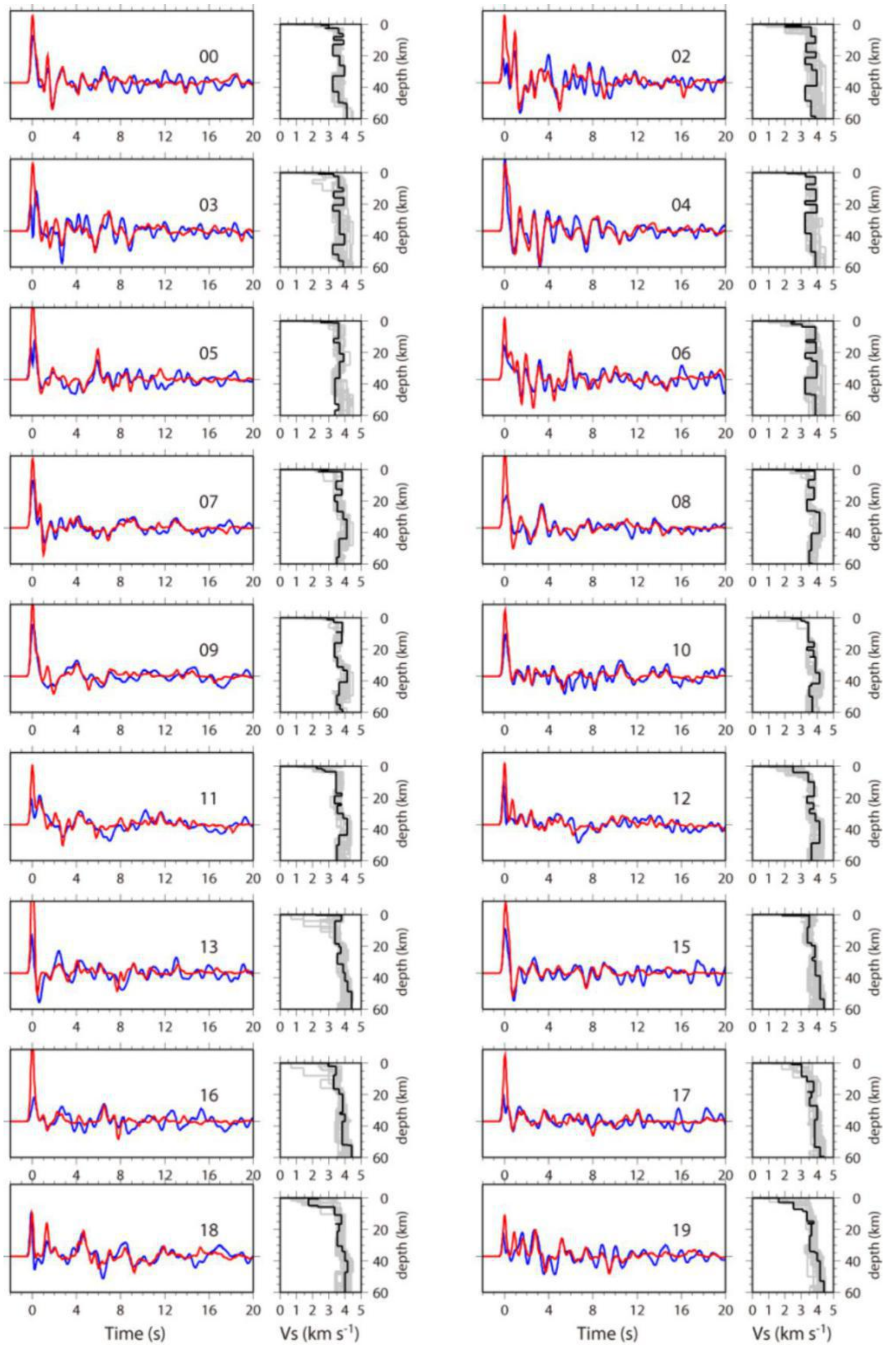

Supplementary Figure 3 Shear wave velocity models and waveform comparisons between synthetic and observed receiver functions for all 38 stations

For each station, the observed (blue lines) and synthetic (red lines) receiver functions are plotted

in the left panel, and the best-fit shear wave velocity models (black lines) are plotted in the right panel. The intermediate results obtained from multiple iteration inversions are shown by gray lines to provide error estimates. The station numbers are given in the left panels.

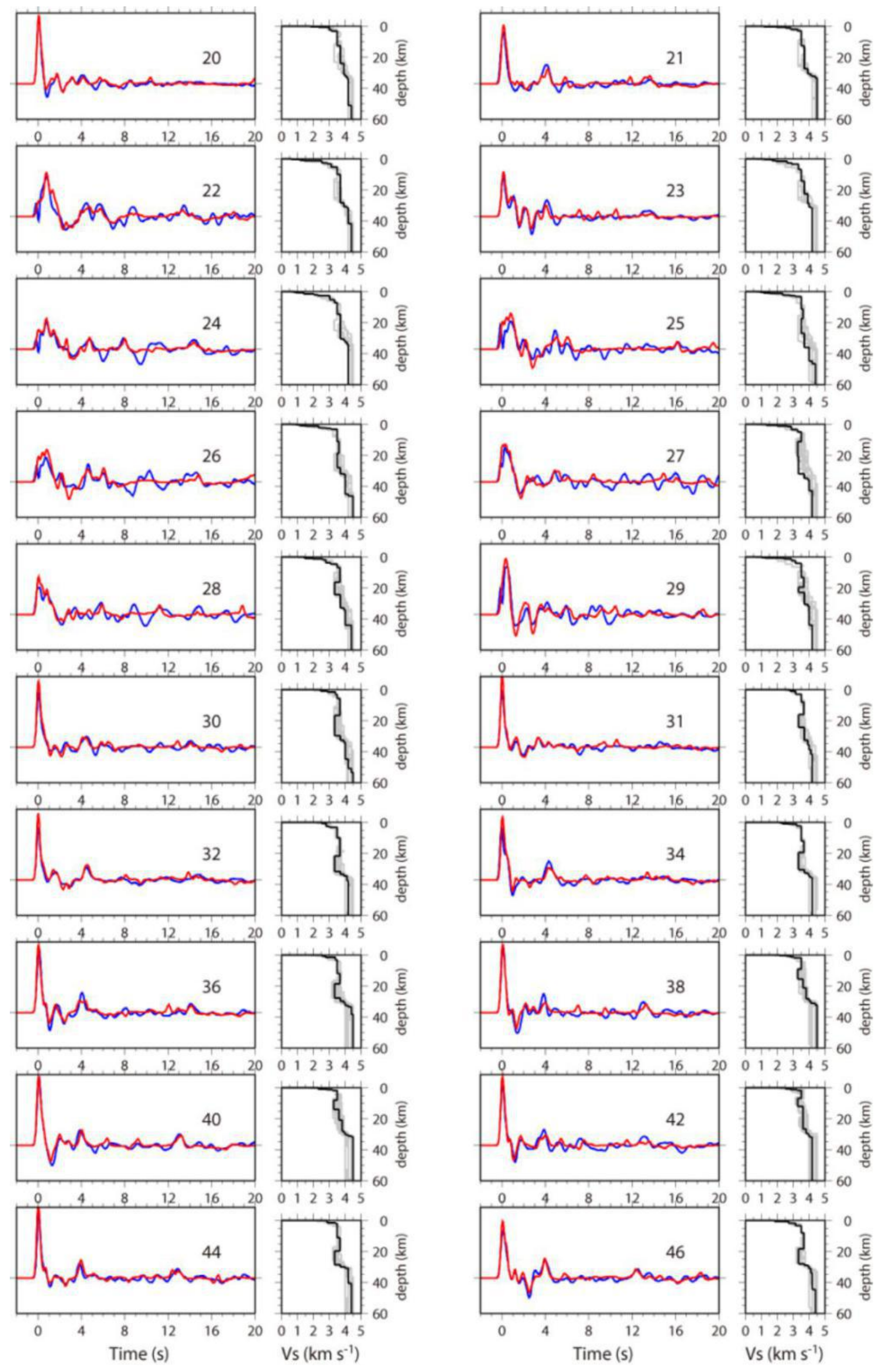

Supplementary Figure 3 (continued)

Supplementary Table 1 Basic information about the seismic stations for which receiver function analysis were performed

Station name	Longitude (E)	Latitude (N)	Location in profile (km)	Number of receiver functions	
				Northern back-azimuth	Southern back-azimuth
M00	93º10'27.93"	22º00'40.81"	38.6	42	76
M02	93º"19"12.29	22º03'43.80"	53.6	40	69
M03	93º24'06.41"	22º03'39.17"	62.0	41	71
M04	93º29'34.70"	22º06'24.61"	71.4	43	71
M05	93º35'41.15"	22º06'47.31"	81.9	44	76
M06	93º38'46.16"	22º05'54.79"	87.2	40	64
M07	93º48'41.37"	22º01'22.98"	104.3	40	70
M08	93º"54"52.76	21º59'06.60"	114.9	22	41
M09	93º59'24.83"	22º02'12.80"	122.7	59	99
M10	94º06'59.54"	22º03'36.78"	135.7	55	75
M11	94º10'58.37"	21º59'52.23"	142.6	24	54
M12	94º13'37.57"	21º59'04.59"	147.2	21	22
M13	94º22'23.33"	22º00'19.22"	162.2	62	101
M15	94º29'03.37"	22º01'55.17"	173.6	38	45
M16	94º33'17.53"	22º02'49.60"	180.9	61	73
M17	94º40'16.88"	22º05'08.15"	192.9	56	95
M18	94º46'57.23"	22º05'23.98"	204.3	57	99
M19	94º52'56.48"	22º24'29.17"	214.5	16	26
M20	94º58'59.25"	22º02'49.16"	225.0	166	
M21	95º04'45.31"	22º06'00.22"	234.8	132	
M22	95º11'39.58"	22º04'45.39"	246.7	108	
M23	95º17'02.55"	22º05'26.51"	255.9	168	
M24	95º24'44.63"	21º59'57.52"	269.3	88	

M25	95º33'08.77"	22º03'09.26"	283.7	54
M26	95º36'40.63"	22º02'39.50"	289.7	85
M27	95º44'46.79"	22º02'39.76"	303.7	54
M28	95º50'31.45"	22º01'11.56"	313.6	153
M29	95º55'50.39"	21º59'59.06"	322.7	130
M30	96º00'14.37"	22º00'35.09"	330.3	123
M31	96º06'22.43"	22º00'50.26"	340.8	42
M32	96º11'39.93"	21º59'58.62"	349.9	142
M34	96º23'26.10"	21º58'26.55"	370.2	163
M36	96º34'24.34"	22º01'57.65"	389.9	139
M38	96º40'50.22"	22º12'04.77"	408.5	170
M40	96º49'40.31"	22º18'47.43"	427.7	137
M42	96º56'22.21"	22º20'59.24"	439.8	108
M44	97º05'00.61"	22º33'07.49"	463.4	139
M46	97º18'50.19"	22º36'04.25"	486.9	110

The value of the number of receiver functions at stations M20-M46 is the total number within all azimuths. The stations were equipped with two kinds of broadband sensors, Nanometrics Trillium 120PA (50 Hz to 120 s) and Guralp CMG 3ESP (50 Hz to 30/60 s) instruments, and a Reftek 130 data acquisition system.

Supplementary Table 2 Estimated dip angle of dipping structures for the resulting and test migration models, and standard deviations and maximum deviations with 90 percent confidence intervals for interface depths and dipping structure thicknesses

Migration model /method	Dip angle (°)	Depth of Moho (km)		Depth of top-interface (km)		Depth of bottom-interface (km)		Thickness of dipping structure (km)	
		Std	Max	Std	Max	Std	Max	Std	Max
**PREM**	18.6	2.6	5.3	5.9	10.5	5.7	7.2	3.9	7.6
**Dipping structure with dip angle of 0°**	19.7			1.3	3.4	0.9	2.6	2.3	5.8
**Dipping structure with dip angle of 10°**	18.8			1.7	4.1	0.9	2.7	2.6	5.3
**Dipping structure with dip angle of 30°**	21.9			2.8	5.5	2.8	4.5	8.1	12.5
**Dipping structure with dip angle of 40°**	20.7			2.5	6.1	2.5	3.3	7.0	9.5
**Best-fit velocity model with a horizontal converter**	18.8			1.5	2.9	1.5	1.1	2.4	4.4

Std: standard deviations; Max: maximum deviations within 90 percent confidence intervals.

Supplementary References

1. Amante, C. and B.W. Eakins. ETOPO1 1 Arc-Minute Global Relief Model: Procedures, Data Sources and Analysis. NOAA Technical Memorandum NESDIS NGDC-24. National Geophysical Data Center, NOAA. doi:10.7289/V5C8276M [access date] (2009).
